# Supplementary material for: Single cell transcriptome analysis of human, marmoset and mouse embryos reveals common and divergent features of preimplantation development
Source: Development. 2018 Nov 9;145(21):dev167833. doi: 10.1242/dev.167833 (PMC6240320; doi:10.1242/dev.167833)
Supplement: Supplementary information [file develop-145-167833-s1.pdf]

Supplementary Figures

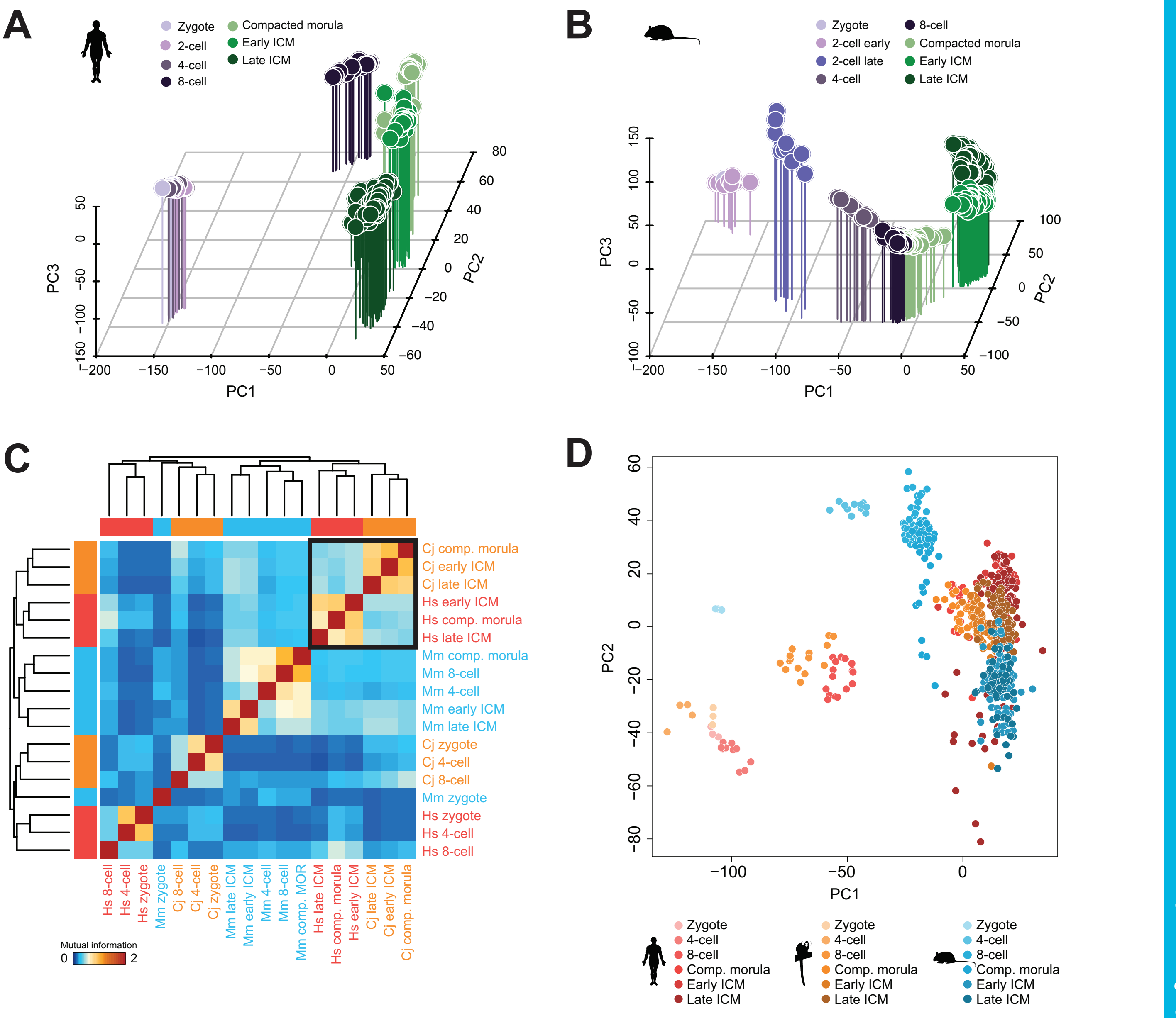

Fig. S1: (A,B) PCA of single-cell embryo samples based on genes detected from minimal transcript coverage (FPKM >0) in human (A) and mouse (B). (C) Clustering of mutual information entropy for orthologous genes co-expressed in human, marmoset and mouse. (D) PCA of samples from all species based on orthologue *Z*-scores.

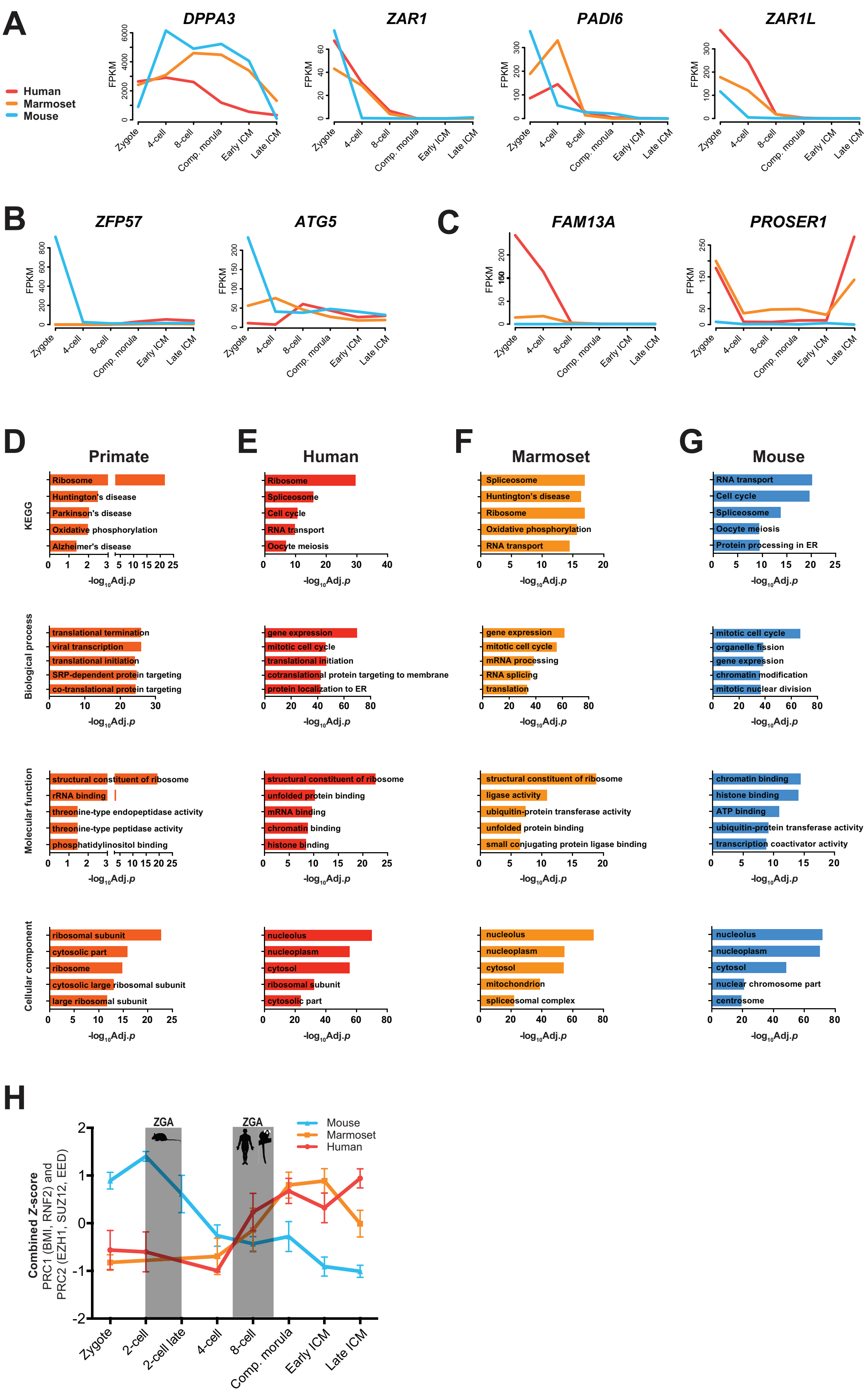

Fig. S2: (A) Transcript abundance of conserved maternal effect genes in human, marmoset and mouse. (B,C) Profile of (B) mouse- and (C) primate-specific maternal effect genes over developmental time in all species. (D–G) Gene ontology (GO) term enrichment analyses for zygotic transcripts (FPKM >10) in (D) primate, (E) human, (F) marmoset and (G) mouse. (H) Combined *Z*-scores of PRC1 and PRC2 components over seven developmental stages including 2-cell embryos.

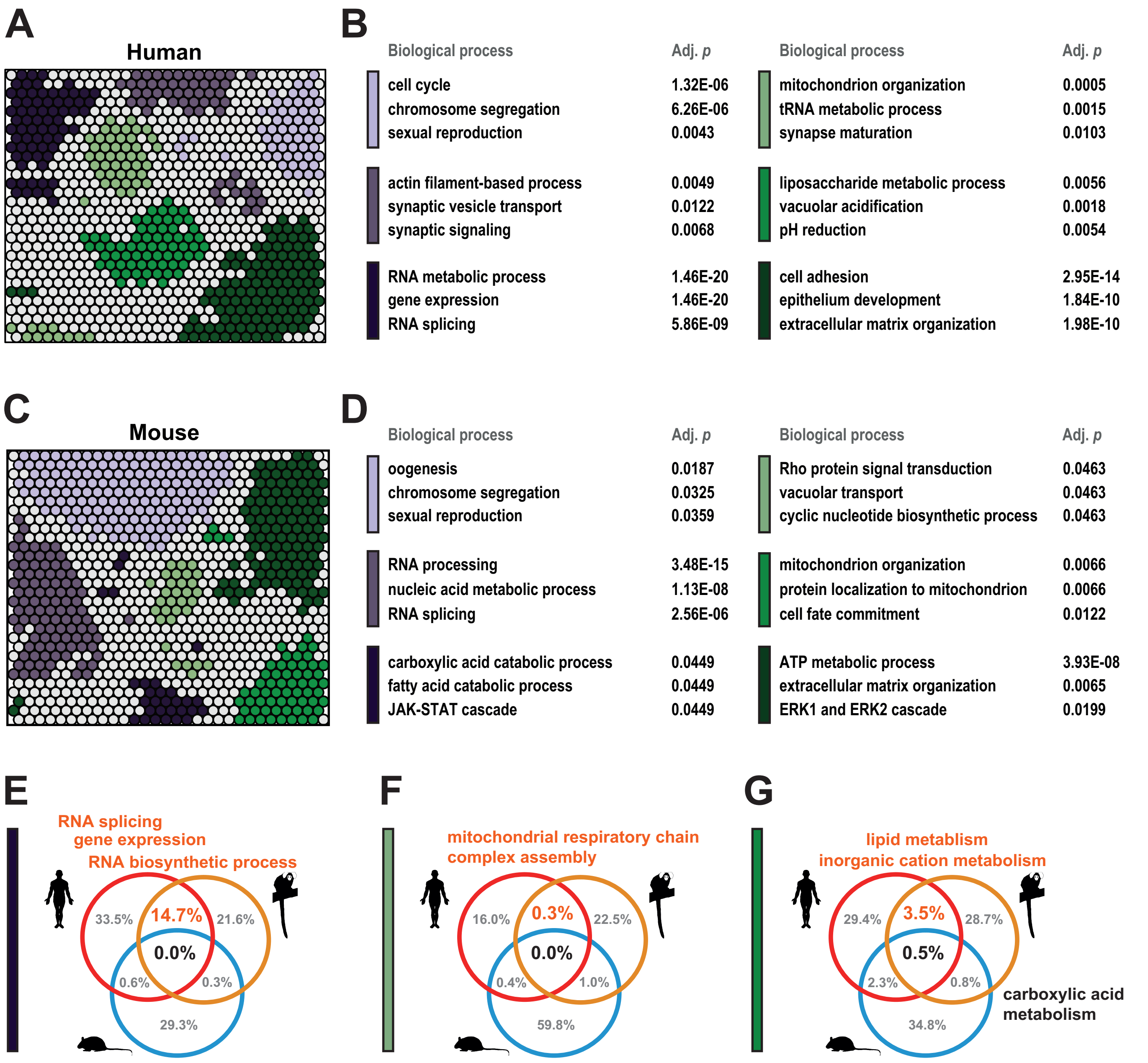

Fig. S3: (A) Self-organizing map (SOM) of human embryo transcriptomes. Clusters (*Z*-score >1.5) are coloured by stage specificity. (B) Biological processes enriched for human SOM clusters. C) SOM of mouse embryo samples. (D) GO processes enriched in mouse SOM clusters. (E–G) Significantly enriched (*p* <0.05) biological processes in human, marmoset and mouse at the 8-cell (E), compacted morula (F) and early ICM (G) stages.

Development • Supplementary information

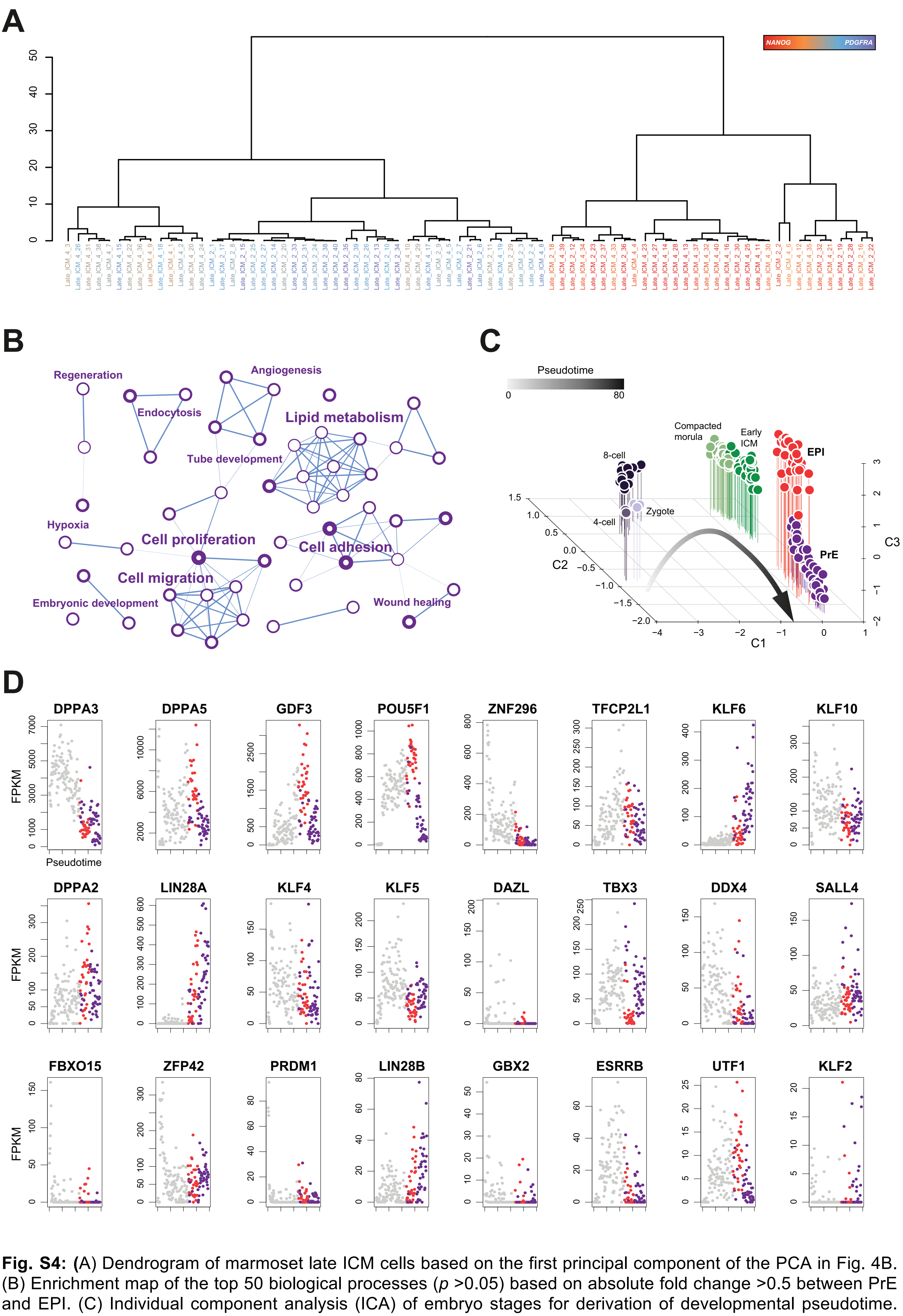

Fig. S4: (A) Dendrogram of marmoset late ICM cells based on the first principal component of the PCA in Fig. 4B. (B) Enrichment map of the top 50 biological processes (*p* >0.05) based on absolute fold change >0.5 between PrE and EPI. (C) Individual component analysis (ICA) of embryo stages for derivation of developmental pseudotime. (D) Absolute expression of selected pluripotency and germ cell markers, ordered by pseudotime.

Development • Supplementary information

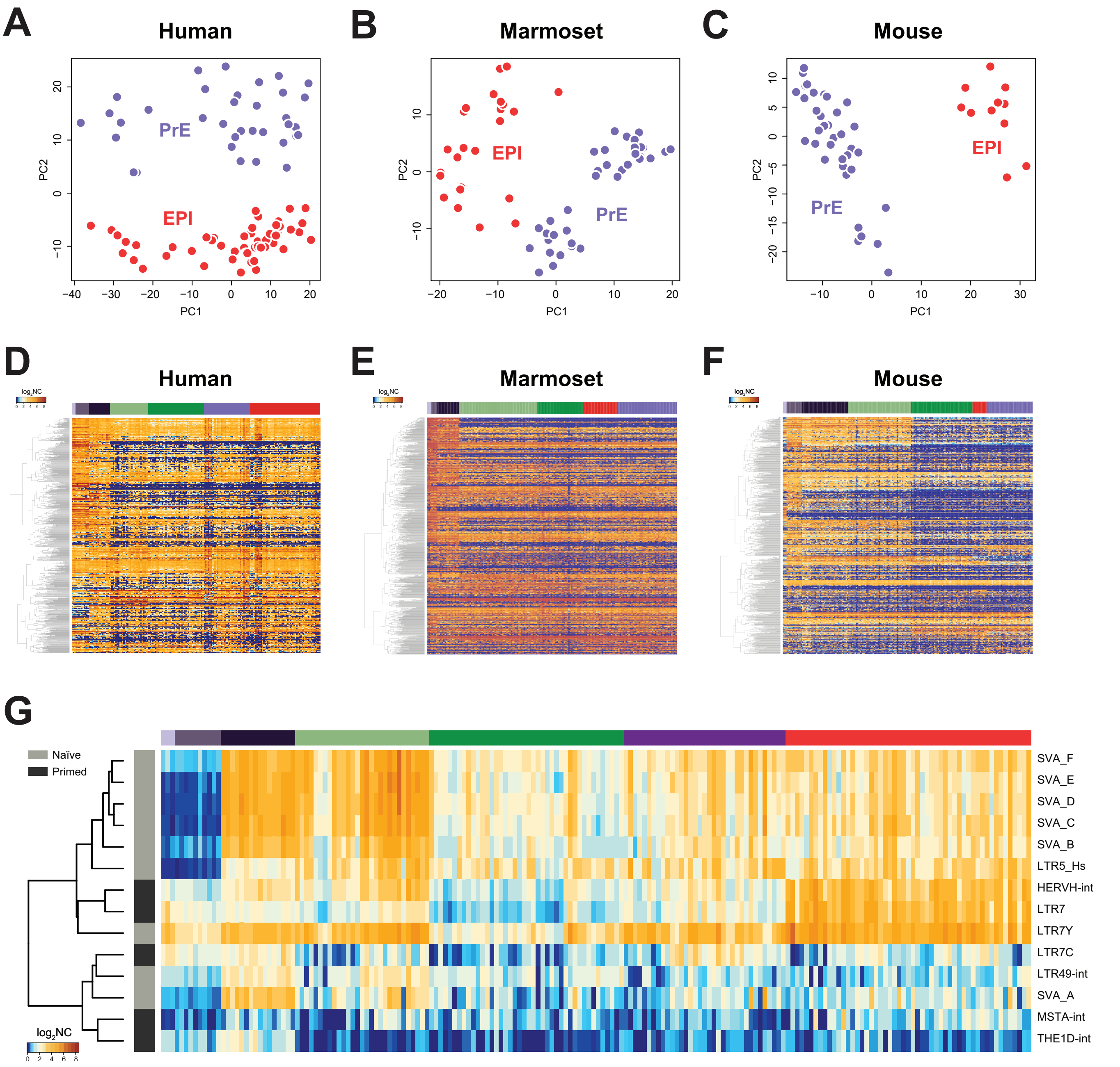

Fig. S5: (A–C) PCA of human (A), marmoset (B) and mouse (C) EPI and PrE cells (log_2 FPKM >0.5 and $logCV^2$ >1). (D–F) One-way hierarchical clustering of averaged expression of transposable element families detected in human (D), marmoset (E) and mouse (F) embryos. (G) One-way hierarchical clustering of averaged expression of transposons associated with naïve and primed human PSC as defined in (Theunissen et al., 2016).

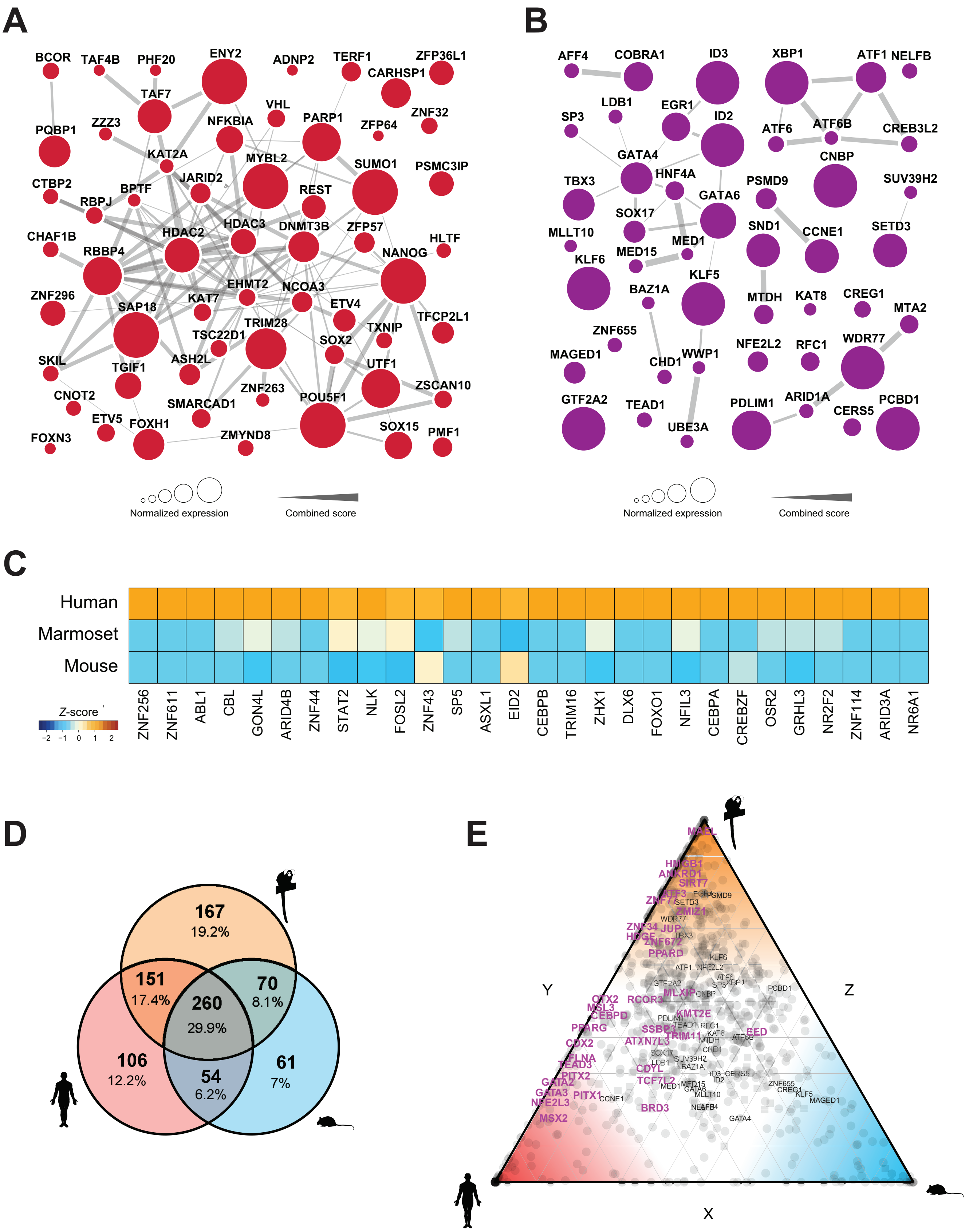

Fig. S6: (A,B) Protein-protein interaction network of conserved EPI (A) and PrE (B) transcription factors in human, marmoset and mouse. Genes in (A) are derived from the analysis in Fig. 6A, and those in (B) from Fig. S6D above. Node sizes are scaled to normalised expression in human and marmoset; edges are derived from the STRING database. (C) Human transcription factors specific to primitive endoderm. (D) Intersection of transcription factors specific to human, marmoset and mouse PrE (FPKM >5 in PrE and not significantly (p >0.05) upregulated in EPI). (E) PrE-enriched transcription factors (circles) and chromatin remodelling factors (squares) between human, marmoset and mouse. Axes show the relative fraction of expression in the EPI between mouse and human (x), human and marmoset (y), and marmoset and mouse (z).

Development • Supplementary information

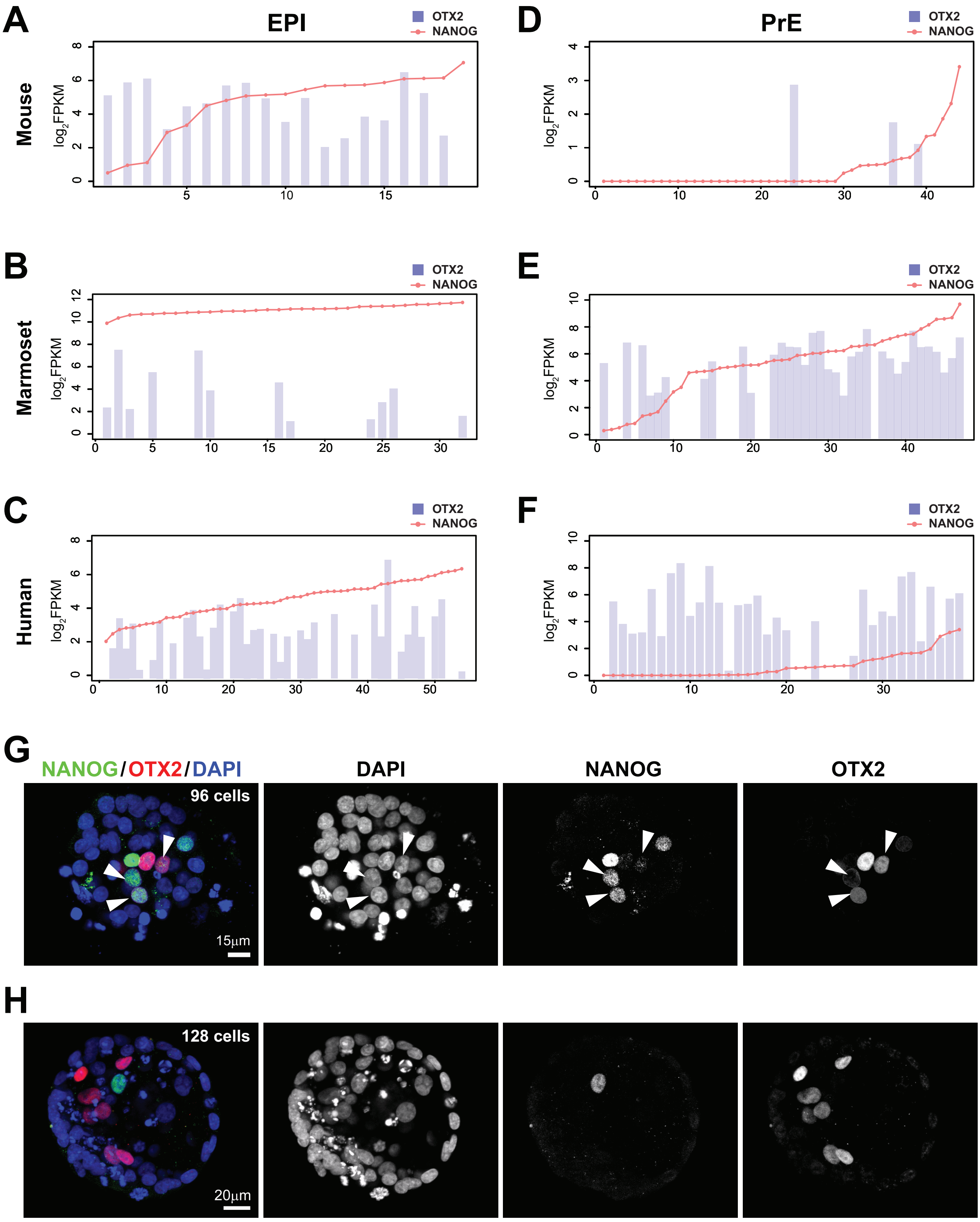

Fig. S7: Plots generated by the web resource showing expression of *OTX2* in relation to *NANOG* in each species. (A–F) *OTX2* and *NANOG* mRNA levels in $\log_2$ FPKM for EPI in mouse (A), marmoset (B) and human (C), and PrE in mouse (D), marmoset (E) and human (F). (G,H) Confocal immunofluorescence sections of NANOG, OTX2 and DAPI in early (G) and late (H) human blastocysts. (G) Co-localisation of NANOG and OTX2 (arrows) is observed in cavitating embryos with intermediate protein abundance. (H) Mutually exclusive expression of NANOG and OTX2 is evident in later-stage embryos.

Development • Supplementary information

Tables

Table S1: Libraries and coverage statistics for single-cell RNA-seq samples from common marmoset embryos, spanning zygote to late preimplantation blastocyst stages.

Click here to Download Table S1

Table S2: Annotated orthologs for human, marmoset and mouse transcriptomes with average expression in preimplantation embryo stages.

Click here to Download Table S2

Table S3: Differential expression of genes detected in EPI and PrE lineages for human, marmoset and mouse.

Click here to Download Table S3

Table S4: Average gene expression in six preimplantation embryo stages for human, marmoset and mouse.

Click here to Download Table S4

Table S5: Average expression of transposable elements annotated by family and class in six preimplantation embryo stages including EPI and PrE for human, marmoset and mouse.

Click here to Download Table S5

Table S6: Average gene expression in six preimplantation embryo stages including EPI and PrE for human, marmoset and mouse, annotated for transcription factors, co-factors and chromatin modifiers.

Click here to Download Table S6

Table S7: Classes and families of transposable elements for human, marmoset and mouse.

Click here to Download Table S7
